# Genome Sequence of Litorilinea aerophila, an Icelandic Intertidal Hot Springs Bacterium

**DOI:** 10.1128/MRA.01206-21

**Published:** 2022-01-27

**Authors:** Elizabeth G. Maurais, Lauren C. Iannazzi, Kyle S. MacLea

**Affiliations:** a Biotechnology Undergraduate Program, University of New Hampshire, Manchester, New Hampshire, USA; b Biology Undergraduate Program, University of New Hampshire, Manchester, New Hampshire, USA; c Graduate Program in Biotechnology: Industrial and Biomedical Sciences, University of New Hampshire, Manchester, New Hampshire, USA; d Department of Life Sciences, University of New Hampshire, Manchester, New Hampshire, USA; Montana State University

## Abstract

The hot springs bacterium Litorilinea aerophila PRI-4131^T^ (= ATCC BAA-2444^T^) was found in Isafjardardjup, in northwest Iceland. In this paper, we present a draft genome sequence for the type strain, with a total predicted genome length of 6,043,010 bp, 4,608 protein-coding sequences, 54 RNAs, 9 CRISPR arrays, and a G+C content of 64.61%.

## ANNOUNCEMENT

The bacterial phylum *Chloroflexi* (1–3) (also *Chlorobacteria* or *Chloroflexota*) is a deep-branching bacterial phylum with significant metabolic diversity, from green non-sulfur photosynthesizers to anaerobic halogen metabolizers to aerobic chemoorganotrophs (4–9). Thermophilic growth is common. The *Chloroflexi* are unusual in that cells stain as Gram negative but possess a single cell wall layer (i.e., they are monoderms) with no evidence of an outer membrane, which is characteristic of most other Gram-negative bacteria (2, 3, 10–12).

Within the *Chloroflexi* class *Caldilineae*, most organisms are anaerobes, but two of these filamentous thermophilic bacteria, including Litorilinea aerophila, have shown aerobic growth (4, 9). In this work, we describe a draft genome sequence for the type strain of Litorilinea aerophila, PRI-4131 (= DSM 25763 = ATCC BAA-2444). Litorilinea aerophila was isolated from an intertidal hot spring (0.6% NaCl) in Iceland (9) but has since been found in other environments, including in the human cervicovaginal microbiota (13), in waste treatment and disposal sites/systems (14–16), in plant cultivation systems (17), and in mines (18).

Lyophilized Litorilinea aerophila ATCC BAA-2444^T^ was purchased from ATCC (Manassas, VA, USA), resuspended in marine broth 2216 (BD, Franklin Lakes, NJ, USA), and incubated at 50°C for 5 days at 1 atm. It was then subcultured on marine agar (5 days at 50°C), from which a single colony was inoculated into 2 mL marine broth. After growth at 50°C to log phase, genomic DNA (gDNA) was purified using the QIAamp DNA minikit (Qiagen, Valencia, CA, USA). Fragmentation of gDNA and adapter attachment were performed using the KAPA HyperPlus kit v.3.16 (KR1145; Kapa Biosystems, Wilmington, MA, USA). An Illumina HiSeq 2500 instrument (Hubbard Center for Genome Studies, Durham, NH, USA) was used for paired-end 250-bp fragment sequencing. Reads were trimmed using Trimmomatic v.0.38 (settings: paired-end mode with a window size of 4, quality requirement of 15, and minimum read length of 36), and then 1,522,708 trimmed reads were assembled with SPAdes v.3.13.0 (19, 20) with default bacterial assembly parameters. Small contigs (<500 bp) and contigs with low coverage (<10×) were removed. QUAST (21) analysis of this assembly showed 93 contigs (the largest one being 341,248 bp), with an *N*_50_ value of 180,513 bp. Benchmarking universal single-copy orthologs (BUSCO) v.5.2.2 analysis (with default parameters) showed that the assembly was 95.2% complete (22), and genome coverage of 112× was calculated. The NCBI Prokaryotic Genome Assembly Pipeline (PGAP) (23) identified and annotated genes in the L. aerophila genome. The assembled genome was 6,043,010 bp in length, and PGAP revealed a total of 4,749 genes, 4,608 protein-coding sequences, 87 pseudogenes, 46 tRNAs, 5 partial or complete copies of the rRNA genes, including 1 complete copy of each, 3 noncoding RNAs, and a G+C content of 64.61%, close to the published value of 64.7% for the species (9). Nine CRISPR arrays were identified, as well as the CRISPR-associated genes encoding Cas1 to Cas3 and Cas5e (24). As predicted based on analysis of the *Chloroflexi* (11, 12), *Litorilinea* lacks Gram-negative lipopolysaccharide (LPS) and lipid A metabolic genes such as *lpxC* and also possesses teichoic acid and lipoteichoic acid transport and synthesis genes (24), which are characteristic of monoderms.

### Data availability.

The Litorilinea aerophila ATCC BAA-2444^T^ whole-genome shotgun sequencing (WGS) project has been deposited in DDBJ/ENA/GenBank under accession number VIGC00000000. The raw Illumina data from BioProject PRJNA551245 were submitted to the NCBI Sequence Read Archive (SRA) under accession number SRX6432641.
